# Consideration of corneal biomechanics in the diagnosis and management of keratoconus: is it important?

**DOI:** 10.1186/s40662-016-0048-4

**Published:** 2016-07-04

**Authors:** FangJun Bao, Brendan Geraghty, QinMei Wang, Ahmed Elsheikh

**Affiliations:** The Affiliated Eye Hospital of Wenzhou Medical University, Wenzhou City, 325027 China; The Institution of Ocular Biomechanics, Wenzhou Medical University, Wenzhou City, 325027 China; School of Engineering, University of Liverpool, Liverpool City, L69 3GH UK; NIHR Biomedical Research Centre for Ophthalmology, Moorfields Eye Hospital NHS Foundation Trust and UCL Institute of Ophthalmology, London City, UK

**Keywords:** Keratoconus, In vivo, Corneal biomechanics, Corneal collagen cross-linking

## Abstract

Keratoconus is a bilateral, non-inflammatory, degenerative corneal disease. The occurrence and development of keratoconus is associated with corneal thinning and conical protrusion, which causes irregular astigmatism. With the disruption of the collagen organization, the cornea loses its shape and function resulting in progressive visual degradation. Currently, corneal topography is the most important tool for the diagnosis of keratoconus, which may lead to false negatives among the patient population in the subclinical phase. However, it is now hypothesised that biomechanical destabilisation of the cornea may take place ahead of the topographic evidence of keratoconus, hence possibly assisting with disease diagnosis and management. This article provides a review of the definition, diagnosis, and management strategies for keratoconus based on corneal biomechanics.

## Background

Keratoconus (KC) is an idiopathic degenerative eye disease characterised by localized thinning and conical protrusion of the cornea, which typically develops in the inferior-temporal and central zones [1]. Consequently, visual acuity is reduced due to irregular astigmatism and high myopia resulting from asymmetric topographical changes in the anterior corneal surface. KC is the most prevalent form of corneal ectasia and affects all ethnicities [2–5], however, higher incidence has been reported in Asians when compared to Caucasians [6, 7]. While the aetiology and pathology of the disease is still not fully understood, various biochemical, cellular and microstructural differences have been reported in the literature. For instance, biochemical changes include increased activity of proteolytic enzymes and a decrease in their inhibitors [8, 9]. Increased proteoglycan (PG) content and altered distribution PG filaments have also been reported [10]. A progressive reduction in collagen-producing corneal keratocytes has been observed [11] as well as a disruption to the highly organized orthogonal arrangement of collagens [12] that is typically seen in healthy corneas [13, 14]. Further, a decrease in the mean fibril diameter and interfibrillar spacing of individual collagens and undulation of collagen lamellae have been reported [10]. Since biomechanical stability is dependent on regulation and organization of structural components within the cornea, the aforementioned biochemical, cellular and microstructural alterations would be expected to have negative consequences on structural integrity and hence lead to corneal abnormal deformation under intraocular pressure. It is therefore no surprise that experimental studies of ex vivo KC corneas have reported abnormalities in biomechanical response to applied loads when compared to normal corneas [15, 16].

## Reviews

### Keratoconus diagnosis techniques

With the disruption of the collagen network, intraocular pressure-related stress causes a weakened cornea to bulge from its normal shape and become progressively conical. Consequently, corneal topography is the most widely used tool to detect KC [17]. Corneal shape parameters such as thin pachymetry, atypical pachymetry profile, irregular anterior curvature as well as increased posterior surface elevation, have all been used to detect KC at different stages of the disease [17]. While topography analysis is well-suited to characterising KC when clear geometrical changes have occurred in the cornea, its robustness reduces when attempting to assess mild, pathologic cases, especially in subclinical or early KC [17]. However, changes in corneal geometric features are secondary signs of KC whereas the earliest initiating changes would occur within the microstructures and then the biomechanical properties of cornea. Therefore, understanding the cornea’s biomechanical behaviour is important for the detection of subclinical KC, while changes in topography are still insufficient to provide conclusive evidence of KC progression [18]. However, in vivo measurement of corneal biomechanics remains a difficult task at this stage and only two commercially available instruments have been proposed to assist in the diagnosis of KC. These two instruments are summarized below.

### Ocular response analyzer

**Fig. 1:**
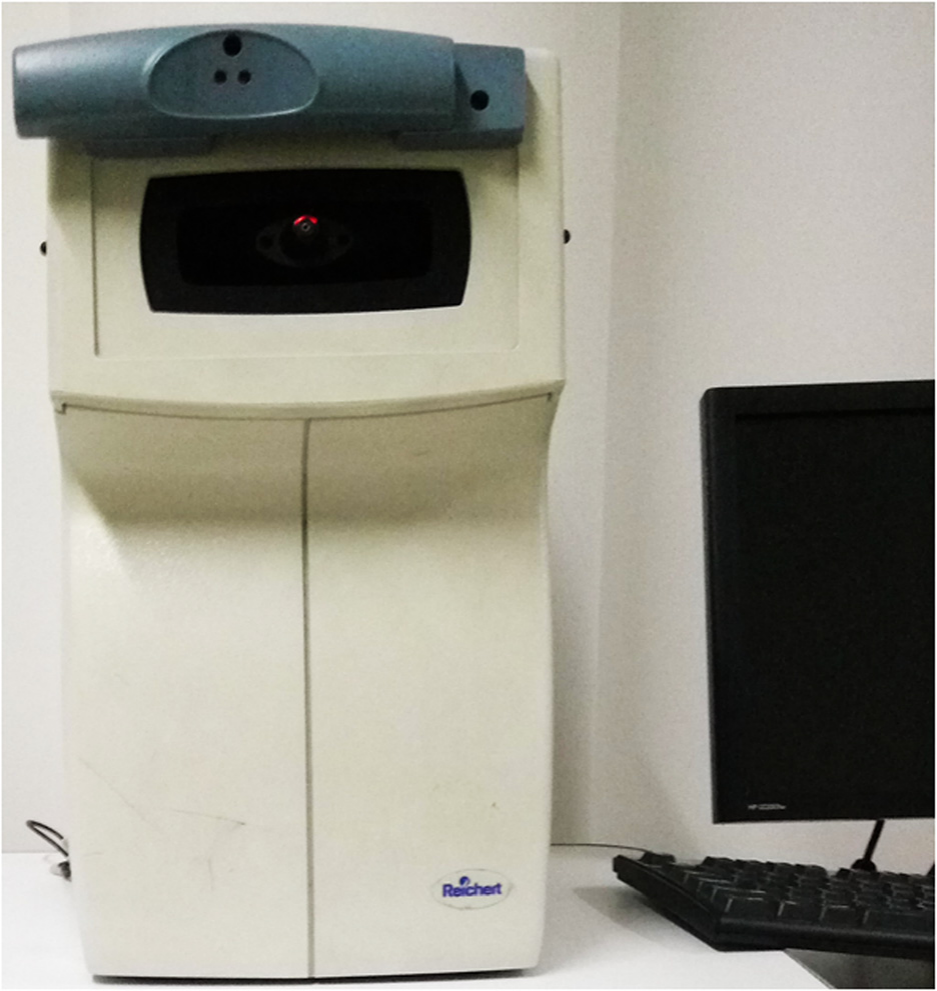
Photo of the Ocular Response Analyzer (ORA)

The ocular response analyzer (ORA) became commercially available in 2005 and was the first device capable of evaluating the biomechanical response of the cornea in vivo (Fig. 1). The device provides two biomechanical metrics: corneal hysteresis (CH) and corneal resistance factor (CRF), both of which are influenced by the viscoelastic behaviour of corneal tissue [19]. Clinically measured metrics provided by the ORA have been widely used to assess the biomechanical response of the cornea. Compared with normal patients, both CH and CRF decrease in KC corneas indicating mechanical softening of the stroma [20]. However, when comparing these biomechanical metrics, it is clear that a wide substantial overlap exists between normal corneas and keratoconic corneas [21, 22] and so they have not been as effective in identifying KC as first anticipated. Furthermore, the exact correlation between these metrics and the established mechanical properties of tissue (such as tangent modulus) is still unknown. Thus, the ORA needs to be complemented with other diagnostic imaging tools to obtain a reliable diagnosis of KC. With the introduction of a new software update (version 2.0) in 2009, the ORA now computes 37 new parameters that describe the waveform of the ORA applanation signal. These parameters show promise in providing additional biomechanical information about the KC cornea [23, 24]. However, specific explanation of the meaning of these parameters has not been provided by the manufacturers, and they still require thorough clinical validation before they can be used clinically.

### Corvis ST

**Fig. 2:**
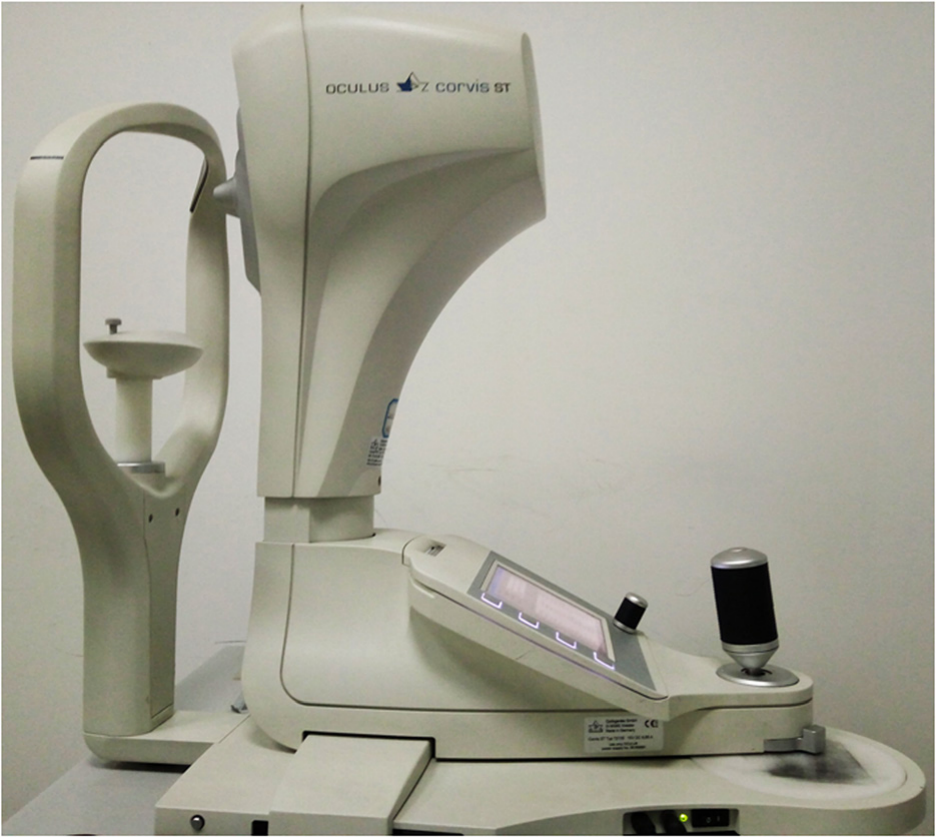
Photo of the Corvis ST (CVS)

The Corvis ST (CVS) is another non-contact device that was introduced in 2010 and provides information about the biomechanical response of the cornea using dynamic Scheimpflug imaging analysis (Fig. 2). The CVS captures approximately 140 cross-sectional images of the cornea during the air-puff induced dynamic deformation [25] using its high-speed camera system. The corneas’ response to air pressure is characterized by ten deformation parameters, some of which are strongly correlated with the tissue’s mechanical stiffness. As shown in a previous study, the maximum deformation amplitude of keratoconic corneas is much greater than that of normal corneas [26]. However, the usefulness of CVS to evaluate KC severity and diagnose subclinical KC is yet to be determined. In addition to the ten metrics provided by the device, the inclusion of a high-speed Scheimpflug camera allows for precise monitoring of cornea cross-sectional deformation under the applied air pressure. The capability to monitor in vivo response of the cornea provides biomedical engineers with essential information that can be used to determine more precise biomechanical properties of the tissue. Work is now progressing to utilise this device to produce regional estimations of in vivo corneal stiffness, which may allow for better planning of the treatment and management of KC.

### Other devices

In addition to the ORA and CVS, several other technologies have been developed to evaluate corneal biomechanical parameters in vivo such as optical coherence tomography [27], supersonic shear wave imaging (SSI) [28], confocal microscopy [29], applanation resonance tonometry (ART) [30], acoustic radiation force (ARF) [31] and scanning acoustic microscopy [32]. However, validation of these technologies in human eyes will be essential before using them to improve the accuracy of KC diagnosis.

The current lack of reliable devices that are capable of characterising true corneal material properties in vivo has meant that the biomechanics of KC have only been investigated to a limited extent. It is now becoming evident that a global biomechanical assessment of the cornea may not be sufficient to fully characterize this typically asymmetric disease. Spatial location of focal weakening in the cornea will be necessary to detect the disease at its early stages as well as fully characterise its progression.

### Keratoconus management techniques

KC is currently managed using a number of methods ranging from contact lenses to intra-stromal corneal ring segment (ICRS) implants and collagen crosslinking (CXL), penetrating keratoplasty (PK) and deep anterior lamellar keratoplasty (DALK). However, the method of management used is dependent on the severity of the ectasia. In early stages, spectacles can correct refractive errors sufficiently. As the disease progresses [2], this method becomes unsuitable for correcting the irregular astigmatism associated with KC. In mild to moderate KC, contact lenses have become the most common and successful method of management providing improved visual acuity whilst decreasing the need for surgical interventions [33]. Although soft lenses provide increased comfort for the wearer, rigid lenses are more prevalent since high levels of irregular astigmatism cannot be corrected with other lens types [34, 35]. Recently, CXL treatment of the cornea is becoming the standard method for halting the progression of the disease at early stages and prevent further deterioration, which may make tissue replacement necessary. PK and more recently, DALK, are used to replace either the entire cornea or 95 % of the stromal layers of the cornea, respectively, with healthy donor tissue in advanced cases of KC that cannot be successfully managed with other methods.

### Contact lenses

Contact lenses aim to improve the anterior curvature of the cornea and increase visual acuity. The lens is held in place by forces generated between the tear film, lens and eye, and several options are currently available for use on keratoconic corneas. Bespoke soft lens options, such as the KeroSoft® lens, are individually lathe cut to fit the specific irregularity of a patients’ cornea resulting in a close fit between the lens and eye. Rigid lenses are made from oxygen permeable material but the degree of fit between the lens and eye varies. Originally, it was hoped that the lens bearing pressure on the cornea could correct or stabilise the ectasia by flattening the cone (flat fit) [36], but this can result in abrasion and scarring of the cornea [37] as well as progression of the cone. In mild KC, an ideal fit can be achieved and the lens is usually intended to rest on the apex of the cone and peripheral cornea (three-point touch). However, as the cone progresses, a compromised fit may need to be accepted as long as it does not cause damage to the cornea [38].

### Intra-stromal corneal ring segments

In contrast to the spectacles and contact lens short-term solutions, which aim to improve visual acuity by improving the anterior curvature of the cornea, more long-term invasive clinical interventions are available. ICRS implants aim to improve the shape of the cornea and halt the progression of the cone. ICRS implants are inserted into the stroma by creating an incision in the peripheral region of the cornea and decrease asymmetrical astigmatism and convexity of the cone [39, 40]. Although there have been no statistically significant differences observed in CH and CRF parameters obtained from the ORA following use of the ICRS [41], the introduction of rigid components to the stroma would be expected to affect the biomechanical behaviour of the tissue. This could be due to tissue scarring within the stroma resulting from the introduction of the ICRS implants or changes in the overall mechanical response of the tissue, particularly in the peripheral region where the implant has been introduced.

### Corneal collagen cross-linking

CXL is commonly achieved by removing the epithelium and saturation of the stroma with riboflavin followed by irradiation of the central region of the cornea using ultraviolet-A light at 365–370 nm [42]. The procedure induces crosslinks between the collagen fibrils and within the proteoglycan-rich coating surrounding them [43] as well as limited linkages among collagen molecules and among proteoglycan core proteins [44]. The outcome of this procedure is an overall increase in mechanical strength [45], which usually halts the progression of the cone [46]. CXL is now fast becoming the most commonly used technique as it can be used to halt the progression of the cone especially at an early stage [47] and with minimum stromal thickness of at least 400 μm, thereby reducing further degradation of visual acuity and limiting the need for corneal transplants.

### Keratoplasty

Advanced cases of KC with high corneal curvature, high astigmatism, low visual acuity, presence of corneal scarring and/or poor contact lens tolerance may require PK [48–50], in which the entire thickness of the cornea is removed and replaced by healthy corneal tissue [2, 48]. However, normal stromal architecture cannot be fully recovered in full-thickness graft wounds [51–53]. On the other hand, if Descemet’s membrane and the endothelium are to remain intact [54, 55], DALK should be a reasonable alternative [56], in which only the stromal layers of the cornea are removed and replaced thereby reducing the risk of endothelial rejection and transplant failure. Nevertheless, long-term abnormalities in collagen fibril orientation and spatial organisation around the entire graft margin have been observed following PK, which may affect corneal biomechanical behaviour and graft stability in the long term [57].

### Biomechanical changes in KC with different management techniques

Although all current KC management techniques involve mechanical interaction with or mechanical changes to the cornea, the design and planning of these interventions do not consider the mechanical properties of the cornea either pre- or post-intervention. For instance, possible hypoxic effects can occur from prolonged lens wear resulting in oedema [58], and hence increased thickness with soft contact lenses even though the materials used have high oxygen permeability. In rigid lens wear, where the interaction between the lens and cornea can be even more pronounced, changes in corneal shape with possible biochemical, cellular and microstructural responses may have subsequent consequences for the overall biomechanical integrity of the cornea. Postoperative ORA assessments have shown that DALK treated corneas return to biomechanical metrics similar to those of normal corneas whereas the corresponding values for PK treated corneas are significantly lower, indicating weaker biomechanical properties [59, 60]. However, the most significant changes observed are those obtained using CXL. Experimental crosslinking studies have reported human corneal stiffness increases in the region of 300 % using riboflavin/UVA treatment [45], but surprisingly no change in inter-laminar cohesion [61]. These changes in corneal stiffness could not be properly validated so far in vivo due to limitations with current clinical biomechanical assessment techniques as discussed in this paper.

## Conclusion

The current inability to measure in vivo corneal biomechanical properties has been a major obstacle in planning and assessing the outcomes of KC interventions. While diagnosis techniques rely on abnormal cornea tomography parameters, changes in corneal geometry are secondary signs of the disease. Consequently, the efficacy of using these parameters is reduced when attempting to assess mild and subclinical cases. Since the earliest changes to KC corneas occur within the microstructure, in vivo assessment of corneal biomechanics may be a more appropriate approach to detecting subclinical KC. The ORA and CVS provide the first step towards the assessment of in vivo of biomechanical properties. In particular, the inclusion of a high-speed Scheimpflug camera on the CVS allows detailed monitoring of the corneas response to an applied air pressure. This invaluable information may be used by biomedical engineers to determine more detailed corneal biomechanical properties and develop a method of identifying the spatial location of focal weakening within the tissue, and hence enable early detection of subclinical KC and optimisation of management techniques to individual patient’s needs.
